# Intradermal immunization by Ebola virus GP subunit vaccines using microneedle patches protects mice against lethal EBOV challenge

**DOI:** 10.1038/s41598-018-29135-w

**Published:** 2018-07-25

**Authors:** Ying Liu, Ling Ye, Fang Lin, Yasmine Gomaa, David Flyer, Ricardo Carrion, Jean L. Patterson, Mark R. Prausnitz, Gale Smith, Gregory Glenn, Hua Wu, Richard W. Compans, Chinglai Yang

**Affiliations:** 1 Key Laboratory of Special Animal Epidemic Disease, Ministry of Agriculture of China, Institute of Special Economic Animals and Plants, Chinese Academy of Agricultural Sciences CAAS, Changchun, Jilin 130112, P. R. China; 2 Emory University School of Medicine, 1518 Clifton Road, Atlanta, GA 30322 USA; 3 Central Laboratory, Tangdu Hospital at the Fourth Military Medical University, Xi’An, 710038 China; 4 Georgia Institute of Technology, 311 Ferst Drive, Atlanta, GA 30332 USA; 5 Novavax Inc., 20 Firstfield Road, Gaithersburg, MD 20878 USA; 6 Texas Biomedical Research Institute, 7620 NW Loop 410, San Antonio, TX 78227 USA

**Keywords:** Infectious diseases, Vaccines

## Abstract

Development of a safe and efficacious filovirus vaccine is of high importance to public health. In this study, we compared immune responses induced by Ebola virus (EBOV) glycoprotein (GP) subunit vaccines via intradermal immunization with microneedle (MN) patches and the conventional intramuscular (IM) injection in mice, which showed that MN delivery of GP induced higher levels and longer lasting antibody responses against GP than IM injection. Further, we found that EBOV GP in formulation with a saponin-based adjuvant, Matrix-M, can be efficiently loaded onto MN patches. Co-delivery of Matrix-M with GP significantly enhanced induction of antibody responses by MN delivery, as also observed for IM injection. Results from challenge studies showed that all mice that received the GP/adjuvant formulation by MN or IM immunizations were protected from lethal EBOV challenge. Further, 4 out of 5 mice vaccinated by MN delivery of unadjuvanted GP also survived the challenge, whereas only 1 out of 5 mice vaccinated by IM injection of unadjuvanted GP survived the challenge. These results demonstrate that MN patch delivery of EBOV GP subunit vaccines, which is expected to enable improved safety and thermal stability, can confer effective protection against EBOV infection that is superior to IM vaccination.

## Introduction

Ebola virus (EBOV) is an enveloped, negative single-stranded RNA virus that belongs to the *Filoviridae* family^1^. Of the 5 species in the *ebolavirus* genus, EBOV has been responsible for 4 of the 5 most serious Ebolavirus outbreaks^2^. In particular, the 2013–2016 epidemic of EBOV in West Africa caused more than 28,600 human infections and over 11,300 deaths^3^. The high fatality rate associated with Ebolavirus infection, and lack of an effective approach for prevention or treatment, signify the importance and urgency of developing an efficacious vaccine strategy to protect against human outbreaks. A number of vaccine strategies have been under development and were shown to protect small laboratory animals with various efficacies^4–6^. Further evaluation of several vaccines for protection against EBOV infection of non-human primates has yielded highly promising results. These include viral-vector based vaccines such as recombinant adenovirus replicons^7^, recombinant VSV^8^, recombinant parainfluenza virus^9^, recombinant Venezuelan equine encephalitis virus replicon particle^10^, and protein-based vaccines such as virus-like particles (VLPs)^11,12^. Of note, studies using different vaccine platforms in NHPs have shown that effective protection correlates closely with vaccine-induced serum antibody levels against the EBOV surface glycoprotein GP^13,14^, underscoring the importance of this response in mediating protection against EBOV infection. In response to the 2013–2016 EBOV epidemic, a number of EBOV vaccine candidates entered clinical trials, most of which are viral vector-based vaccines^3,15^. Notably, a recombinant VSV-based vaccine expressing EBOV GP was used in a ring vaccination Phase III trial in Guinea and shown to be highly efficacious against EBOV infection and transmission^16^, demonstrating that EBOV epidemics can be potentially controlled by vaccination. However, the cold-chain requirement for transportation and storage of the viral vector-based EBOV vaccines poses significant logistical challenges for distribution and application of these vaccines.

Currently, vaccinations are commonly administered via intramuscular (IM) or subcutaneous (SC) injection of vaccines, which are prepared in solution and need to be stored under frozen or refrigerated conditions to maintain their stability. Over the last decade, microneedle (MN) patches have been under development as a new vaccine delivery technology for skin vaccination. MN patches have been fabricated using methods adapted from the microelectronics industry and investigated as novel devices to facilitate intradermal (ID) delivery of drugs and vaccines^17,18^. We and others have previously demonstrated that ID delivery of influenza vaccines using patches containing solid metal MNs or dissolving MNs have the ability to generate potent and effective immune responses equivalent or superior to IM injection, and to protect vaccinated animals against lethal challenge by influenza viruses^19,20^. Further, influenza vaccines in MN patches were found to exhibit superior thermal stability, maintaining their structural integrity and antigenicity after a long period of storage at elevated temperatures^21^. More recently, results from Phase I clinical trials of influenza vaccine showed that immunization by MN patch delivery of influenza vaccines was able to induce robust immune responses in humans that are at least equivalent to vaccination by the conventional IM injection method^22^. Moreover, the results from these studies showed that influenza vaccines in MN patches retained their antigenicity and potency after storage at 5 °C, 25 °C, and 40 °C for up to 12 months, demonstrating the possibility to eliminate the cold-chain requirement for storage and transportation by this vaccine delivery technology.

In addition to viral vector-based EBOV vaccines, an EBOV GP subunit vaccine also entered a Phase I human clinical trial in response to the 2013–2016 EBOV epidemic^15^. This vaccine was based on the wild type full length Makona EBOV GP, which was expressed in Sf9 insect cells with a recombinant baculovirus, and the purified GP trimers were found to self-assemble into spherical particles that are about 36 nm in diameter^23^. The EBOV GP subunit vaccine was further shown to induce protective immune responses against lethal EBOV challenge in mice when applied in formulation with a saponin-based adjuvant Matrix-M by intramuscular (IM) injection. We have previously incorporated an EBOV DNA vaccine into a biodegradable polymer particle formulation which showed improved thermostability and stronger immune responses when delivered by MN patch compared to IM injection^24^. In the present study, we investigated immunogenicity and protective efficacy of the EBOV GP subunit vaccine administered by using MN patches in comparison with the conventional IM injection method. Our results show that EBOV GP subunit vaccines can be successfully coated onto solid metal MN patches, and that immunization by MN patches induced stronger and longer lasting antibody responses against EBOV GP than IM injection. Further, immunogenicity of EBOV GP vaccines on MN patches can be effectively augmented by formulating with the Matrix-M adjuvant as observed previously for IM injection of the EBOV GP nanoparticle vaccines, and can confer complete protection against lethal EBOV challenge.

## Results

### MN patch delivery of EBOV GP subunit vaccines induces stronger and longer lasting antibody responses than IM injection

**Figure 1:**
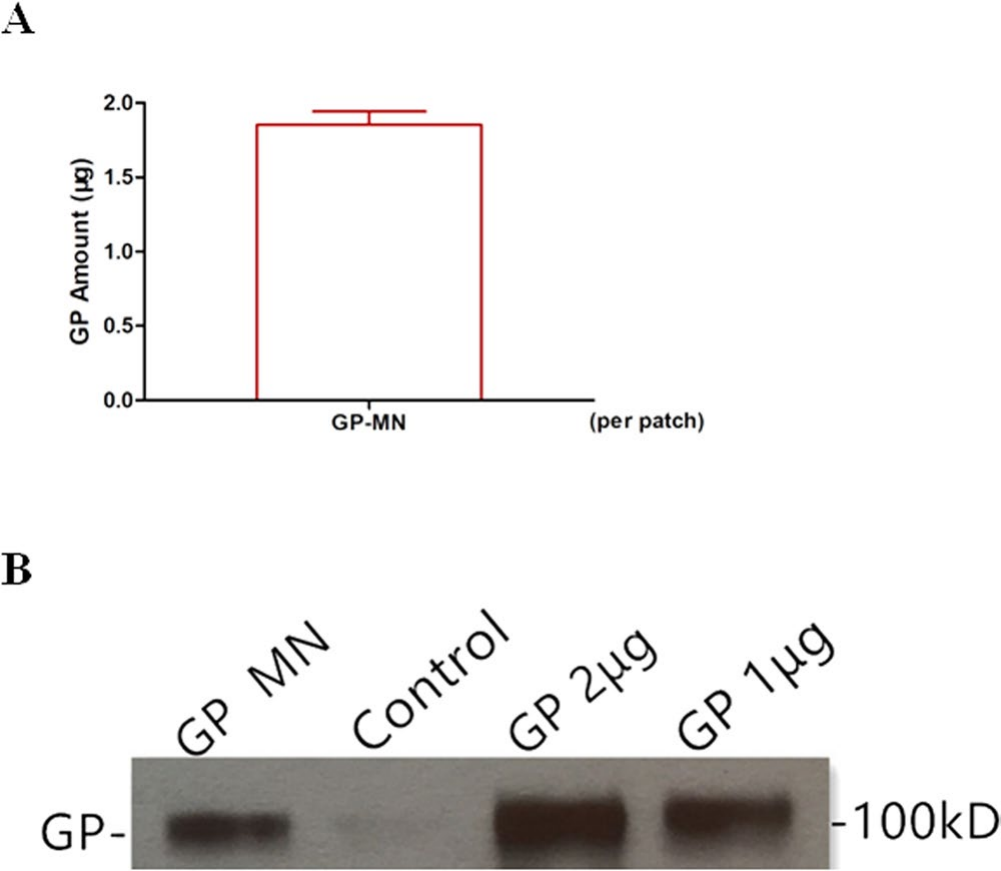
Characterization of GP subunit vaccines coated on MN. GP subunit vaccines were coated onto MN patches as described in Materials and Methods. Five patches were randomly selected from the production batch and GP proteins were dissolved from MN patches by incubating the MN patches in 200 µl PBS, to determine the amount of GP from each MN patch by a quantitative ELISA and to examine GP molecular integrity by Western blot. (**A**) Quantitative ELISA. GP proteins dissolved from each GP-MN were serially diluted and coated onto the wells of a 96-well plate in triplicates. Serial dilutions of purified GP proteins were also coated onto the wells of the 96-well plate at known concentrations. The amount of GP dissolved from GP-MN was determined by ELISA based on a standard curve generated with purified GP nanoparticles with known concentrations. (**B**) Western blot. GP proteins dissolved from GP-MN were concentrated and the protein concentration was determined by a BCA assay. The dissolved GP proteins (1 µg) were analyzed by SDS-PAGE and Western blot in comparison with 1 µg and 2 µg purified GP nanoparticles. The standard curves generated by GP standard in ELISA and the protein bands corresponding to GP are shown and the full-length gel of the Western blot is provided in Supplementary Information (Suppl. Figure 1).

We first determined whether EBOV GP subunit vaccines can be coated onto MN patches. Solid metal MN patches were fabricated and coated with EBOV GP as described in Methods. After fabrication and antigen coating, 5 MN patches were randomly selected, and the GP antigens coated on MN patches were extracted by submerse MN patches in PBS. The quantity of GP coated onto MN patches was then determined by a quantitative ELISA. As shown in Fig. 1A, approximately 1.8 µg of GP were coated onto each MN patch with less than 10% variation, indicating that the EBOV GP subunit vaccines were coated onto MN patches with a high consistency. Further, the GP coated on MN patches were also examined by Western blot in comparison with unprocessed GP, which showed that the process of MN patch production did not affect molecular integrity of GP (Fig. 1B).

**Figure 2:**
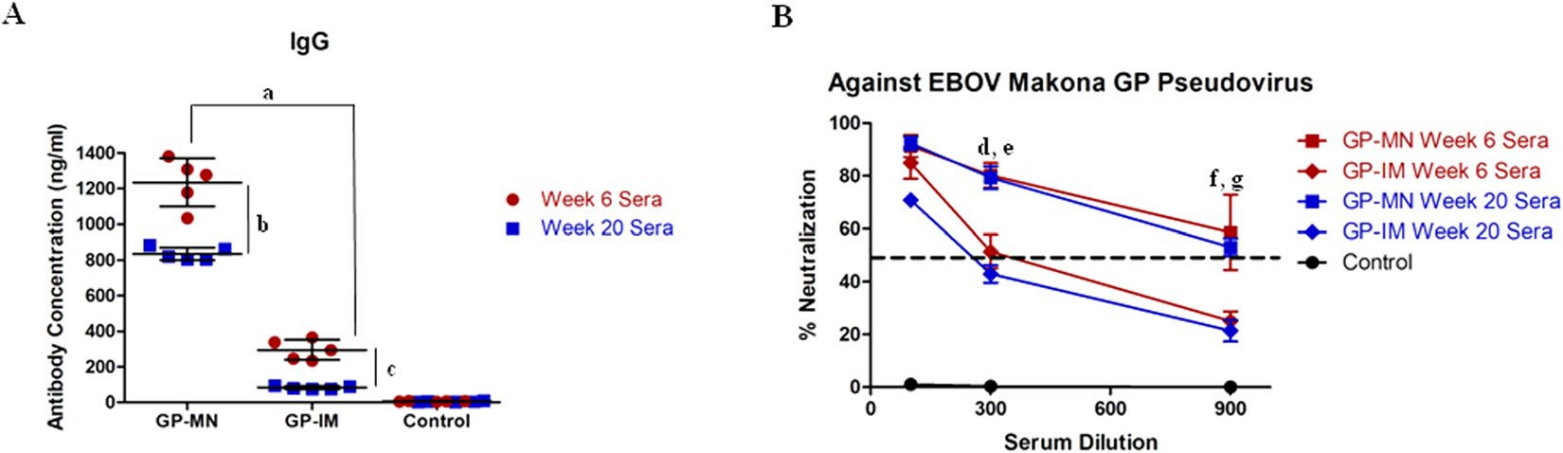
Comparison of antibody responses against GP induced by GP-MN and GP-IM immunizations. Mice (groups of 5) were vaccinated by MN delivery (GP-MN) or IM injection (GP-IM) of 5 µg GP nanoparticle vaccines twice at 4-week intervals. The control group mice received IM injection of 50 µl PBS. Serum samples were collected at 2 (Week 6) and 16 weeks (Week 20) after the second immunization. (**A**) The levels of GP-specific IgG antibodies were determined by ELISA using purified GP as coating antigen. The antibody concentration was determined from a standard curve and expressed as ng/ml anti-GP antibodies. (**B**) Neutralizing activity of sera was determined by incubating 5 × 10^2^ pfu of GP-pseudotyped virus with serial 3-fold dilutions of serum samples from each vaccinated mouse collected at peak and memory phases. Neutralization was measured as percentage decrease in luciferase expression compared to virus-naive mouse sera controls after 48 hours. Statistical analysis for differences between indicated groups (denoted by “a” through “g”) were done by a a two-tailed unpaired t-test. a, GP-MN vs. GP-IM (Week 6), p = 0.0001; b, GP-MN (Week 6) vs. GP-MN (Week 20), p = 0.0055; c, GP-IM (Week 6) vs. GP-IM (Week 20), p = 0.0014; d, GP-MN (Week 6) vs. GP-IM (Week 6) at 1:300 dilution, p = 0.0355; e, GP-MN (Week 20) vs. GP-IM (Week 20) at 1:300 dilution, p = 0.011; f, GP-MN (Week 6) vs. GP-IM (Week 6) at 1:900 dilution, p = 0.0837; d, GP-MN (Week 20) vs. GP-IM (Week 20) at 1:900 dilution, p = 0.0134.

To investigate whether GP subunit vaccines coated onto MN patches retain their immunogenicity, we vaccinated mice with GP-MN patches in comparison with IM injection of GP subunit vaccines at the same dose (5 µg), and analyzed vaccine-induced antibody responses against GP. As shown in Fig. 2A, at two weeks after the second immunization (week 6), immunization by GP-MN induced 4-fold higher levels of antibody against GP compared to IM injection of GP (p = 0.0001). To determine whether antibody responses induced by GP-MN will last over time, blood samples were collected again from the same groups of mice at 16 weeks after the second immunization for analyses (week 20). As also shown in Fig. 2A, antibody levels against GP in sera from GP-MN vaccinated mice remained at high levels, dropping by about 30% from their peak levels (p = 0.0055). In comparison, antibody levels against GP in GP vaccinated mice by IM injection declined by more than 70% (p = 0.0014). Sera collected from vaccinated mice were also analyzed for their neutralizing activity against EBOV-Makona GP-mediated pseudovirus infection, which is the same virus strain used for GP subunit vaccine production. As shown in Fig. 2B, both Week 6 sera and Week 20 sera from GP-MN vaccinated mice exhibited higher levels of neutralizing activity against EBOV-Makona GP pseudoviruses than the serum samples from GP-IM vaccinated mice collected at these time points. Statistical analysis showed that significant differences in neutralizing activity were detected at 1:300 but not 1:900 serum dilutions between Week 6 GP-MN and GP-IM sera (p = 0.0355 at 1:300 serum dilution, and p = 0.0837 at 1:900 serum dilution), whereas significant differences were detected between Week 20 GP-MN and GP-IM sera at both 1:300 and 1:900 serum dilutions (p = 0.011 at 1:300 serum dilution, and p = 0.0134 at 1:900 serum dilution). These results showed that MN delivery of GP induced higher levels and longer lasting antibody responses against EBOV GP than the conventional IM injection.

### MN delivery of adjuvanted GP subunit vaccines augments induction of antibody responses and stimulation induction of IgG2a antibodies

**Figure 3:**
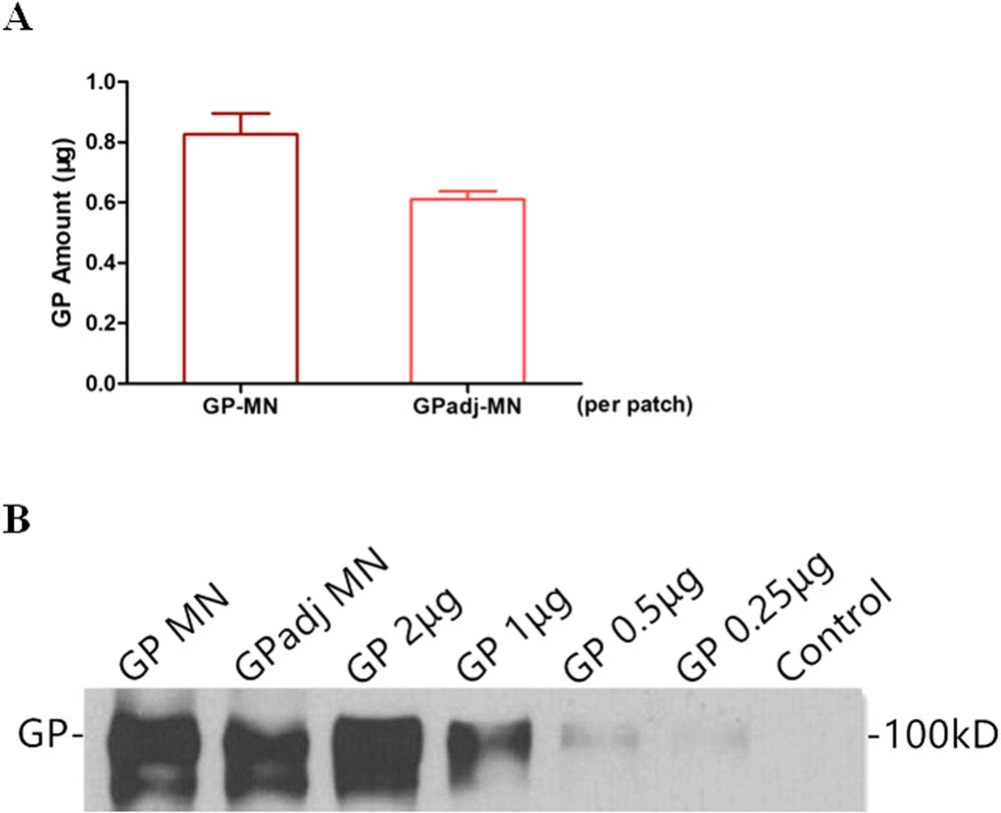
Characterization of GP subunit vaccines in formulation with Matrix-M coated on MN. GP subunit vaccines were formulated with Matrix-M adjuvant at a 1:1 ratio (amount of GP vs. amount of saponin) and then coated onto MN patches as described in Materials and Methods. GP-MN were prepared by mixing GP with the same volume of PBS prior to coating onto MN patches. Five patches were randomly selected from the production batch and GP proteins were dissolved from GPadj-MN and GP-MN patches by soaking the patches in 200 µl PBS, to determine the amount of GP from each MN patch by a quantitative ELISA and to examine GP molecular integrity by Western blot. (**A**) Quantitative ELISA. GP proteins dissolved from each GPadj-MN or GP-MN were serially diluted and coated onto the wells of a 96-well plate in triplicates. Serial dilutions of purified GP were also coated onto the wells of the 96-well plate at known concentrations. The amounts of GP dissolved from GPadj-MN and GP-MN were determined by ELISA based on a standard curve generated with purified GP with known concentrations. (**B**) Western blot. GP proteins dissolved from GPadj-MN or GP-MN were concentrated and the protein concentration was determined by a BCA assay. The dissolved GP proteins (1 µg) were analyzed by SDS-PAGE and Western blot in comparison with 0.25 µg, 0.5 µg, 1 µg, and 2 µg purified GP proteins. The standard curves generated by GP standard in ELISA and the protein bands corresponding to GP are shown and the full-length gel of the Western blot is provided in Supplementary Information (Suppl. Figure 2).

We further investigated whether immune responses induced by MN-GP subunit vaccines can be augmented by an adjuvant, Matrix-M, which is a saponin-based ISCOMATRIX adjuvant developed by Novavax^25^. First, we determined whether GP subunit vaccines in formulation with the Matrix-M adjuvant can be coated onto MN patches. MN patches were dip-coated with GP subunit vaccines in formulation with Matrix-M at a 1:1 ratio, and are designated as GPadj-MN. For comparison, GP-MN patches were produced by coating MN patches with GP subunit vaccines in formulation with PBS at 1:1 ratio. As shown in Fig. 3A, GPadj-MN contained approximately 0.6 µg GP per patch, while GP-MN contained approximately 0.8 µg GP per patch. It is noted that the amount of GP coated on MN patches in this production batch is lower than the previous production batch (Fig. 1A), suggesting that dilution of GP during MN coating may exert a significant effect on coating of GP on MN patches. Nonetheless, the amount of GP on each MN patch varied by less than 10% for both GPadj-MN and GP-MN patches. Further, GP from GPadj-MN and GP-MN patches showed similar migration patterns in Western blot analysis as untreated GP subunit vaccines (Fig. 3B). These results indicate that GP subunit vaccines in formulation with the Matrix-M adjuvant were coated onto MN patches with similar efficiency as unadjuvanted GP subunit vaccines at the same vaccine concentrations.

**Figure 4:**
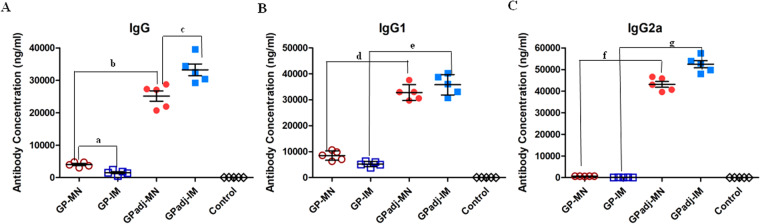
Analysis of antibody responses induced by MN or IM delivery of GP subunit vaccines with or without Matrix-M adjuvant. Mice (groups of 5) were vaccinated twice at 4-week intervals by MN delivery (GPadj-MN) or IM injection (GPadj-IM) of GP subunit vaccines in formulation with Matrix-M, or by MN delivery (GP-MN) or IM injection (GP-IM) of the same amount (5 µg) unadjuvanted GP subunit vaccines. The control group received IM injection of 50 µl PBS. Serum samples were collected at 2 weeks after the second immunization, and the levels of GP-specific total IgG (**A**), IgG1 (**B**), and IgG2a (**C**) antibodies were determined by ELISA using purified GP as coating antigen. The antibody concentration was determined from a standard curve and expressed as ng/ml anti-GP antibodies. Statistical analysis for differences between indicated groups (denoted by “a” through “g”) were done by a a two-tailed unpaired t-test. a, GP-MN vs. GP-IM (IgG), p = 0.0022; b, GPadj-MN vs. GP-MN (IgG), p = 0.0003; c, GPadj-IM vs. GPadj-MN (IgG), p = 0.0552; d, GPadj-MN vs. GP-MN (IgG1), p = 0.0001; e, GPadj-IM vs. GP-IM (IgG1), p = 0.0001; f, GPadj-MN vs. GP-MN (IgG2a), p = 0.0001; g, GPadj-IM vs. GP-IM (IgG2a), p = 0.0001.

Immunogenicity of GPadj-MN and GP-MN vaccines was evaluated in mice and compared with IM injection of GP subunit vaccines with or without the Matrix-M adjuvant, and blood samples were collected at 2 weeks after the second immunization (Week 6) and analyzed for antibody responses against GP. As shown in Fig. 4A, immunization with GP-MN induced higher levels of antibody responses against GP than IM injection of GP (GP-IM) without adjuvant (p = 0.0022). Further, antibody responses against GP induced by GPadj-MN increased by about 8-fold when compared with GP-MN vaccines (p = 0.0003). Similarly, formulation of GP subunit vaccines with Matrix-M adjuvant also greatly augmented induction of antibody responses against GP by IM injection (GPadj-IM), to levels that are comparable to those induced by GPadj-MN (p = 0.0552). Further analysis of the IgG subtypes induced by adjuvanted or unadjuvanted GP subunit vaccines showed that without adjuvant, the antibodies against GP in sera from GP-MN and GP-IM vaccinated mice are primarily of the IgG1 subtype (Fig. 4B), and formulation with the Matrix-M adjuvant increased the levels of IgG1 antibodies against GP by about 4-fold in sera from GPadj-MN or GPadj-IM vaccinated mice (p = 0.0001). Notably, no IgG2a antibodies against GP were induced by unadjuvanted GP subunit vaccines (Fig. 4C). In contrast, immunization with adjuvanted GP subunit vaccines either by MN patches or by IM injection induced high levels of IgG2a antibodies (p < 0.0001). These results show that formulation of Matrix-M adjuvant with GP not only drastically increased the levels of antibody responses against EBOV GP but also changed the profiles of the antibody responses from an IgG1 dominated response to an IgG1/IgG2a balanced response.

### Immunization with adjuvanted GP subunit vaccines by MN patches or IM injection augments induction of neutralizing antibodies that target epitopes outside of the mucin-like domain in GP

**Figure 5:**
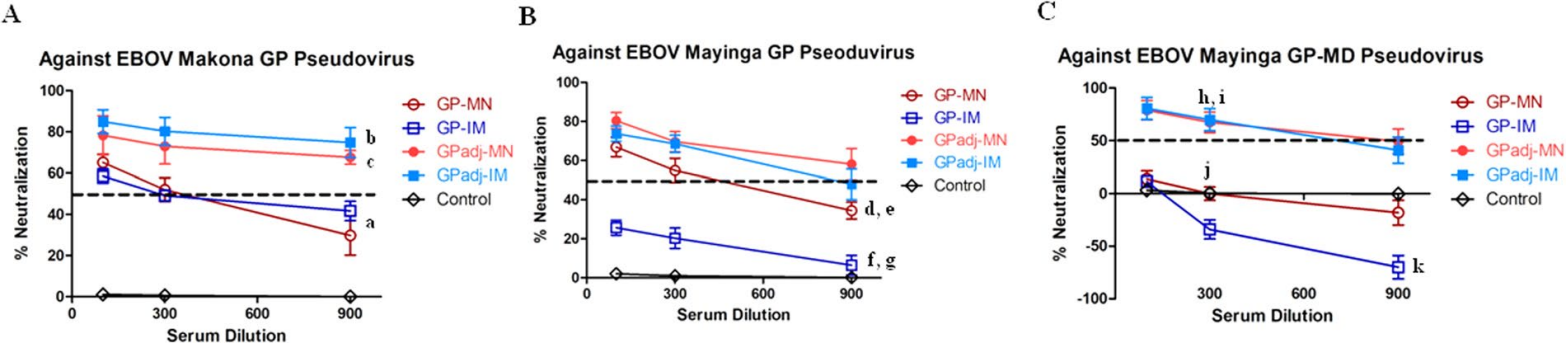
Neutralization of EBOV-Makona GP, EBOV-Mayinga GP, and EBOV-Mayinga GP-MD pseudovirus infection. Mice (groups of 5) were vaccinated twice at 4-week intervals by MN delivery (GPadj-MN) or IM injection (GPadj-IM) of GP subunit vaccines in formulation with Matrix-M, or by MN delivery (GP-MN) or IM injection (GP-IM) of the same amount (5 µg) unadjuvanted GP subunit vaccines. The control group mice received IM injection of 50 µl PBS. Serum samples were collected at 2 weeks after the second immunization. Neutralizing activity of sera was determined by incubating 5 × 10^2^ pfu of Makona EBOV GP (**A**), Mayinga EBOV GP (**B**), or mucin-deleted Mayinga EBOV GP-MD (**C**) pseudotyped viruses with serial 3-fold dilutions of serum samples from each vaccinated mouse. Neutralization was measured as percentage decrease in luciferase expression compared to virus-naive mouse sera controls after 48 hours. Statistical analysis for differences between indicated groups (denoted by “a” through “g”) were done by a a two-tailed unpaired t-test. a, GP-MN vs. GP-IM (1:900 dilution), p = 0.2561; b, GPadj-IM vs. GP-IM (1:900 dilution), p = 0.0329; c, GPadj-MN vs. GP-MN (1:900 dilution), p = 0.0343; d, GPadj-MN vs. GP-MN (1:900 dilution), p = 0.0661; e, GPadj-IM vs. GP-MN (1:900 dilution), p = 0.1679; f, GPadj-MN vs. GP-IM (1:900 dilution), p = 0.0161; g, GPadj-IM vs. GP-IM (1:900 dilution), p = 0.0242; h, GPadj-MN vs. Control (1:300 dilution), p = 0.0073; i, GPadj-IM vs. Control (1:300 dilution), p = 0.009; j, GP-MN vs. Control (1:300 dilution), p = 0.1915; k, GP-IM vs. Control (1:900 dilution), p = 0.0108.

Serum samples from vaccinated mice were further analyzed for their neutralizing activity against EBOV GP pseudoviruses. As shown in Fig. 5A, sera from mice immunized with unadjuvanted GP subunit vaccines by either MN patches or IM injection neutralized EBOV-Makona GP pseudoviruses at similar levels (p = 0.2561 at 1:900 dilutions), with neutralizing activities dropping below 50% at 1:900 serum dilutions. In comparison, neutralizing activity of sera from mice immunized with adjuvanted GP nanoparticle vaccines by either MN patches or IM injection showed higher levels of neutralizing activity against EBOV-Makona GP pseudoviruses, neutralizing over 70% of pseudoviruses at 1:900 serum dilutions (p = 0.0329 for GPadj-IM vs. GP-IM, and p = 0.0343 for GPadj-MN vs. GP-MN). These results showed that formulation with the Matrix-M adjuvant augmented induction of neutralizing antibodies by MN delivery of GP subunit vaccines similarly to results with IM injection. Sera from vaccinated mice were also analyzed for their neutralizing activity against pseudoviruses with EBOV-Mayinga GP. As shown in Fig. 5B, sera from GPadj-MN and GPadj-IM -vaccinated mice were able to neutralize about 50% of EBOV-Mayinga GP pseudoviruses at 1:900 dilutions, whereas sera from GP-MN vaccinated mice neutralized about 35% EBOV-Mayinga GP pseudoviruses at 1:900 dilutions, and the differences between these groups are not significant (p > 0.05). However, sera from GP-IM vaccinated mice only neutralized about 20% of EBOV-Mayinga GP pseudoviruses at 1:100 dilutions, which is significantly lower than other groups (p < 0.01). EBOV-Mayinga GP differs from EBOV-Makona GP by 20 amino acids, with the majority of the differences (15 amino acids) located in the mucin-like domain. We further investigated neutralizing activity of sera from vaccinated against pseudovirus infection mediated by a mucin-like domain deleted EBOV-Mayinga GP, designated as EBOV-Mayinga GP-MD. As shown in Fig. 5C, sera from GPadj-MN and GPadj-IM vaccinated mice were able neutralize over 50% EBOV-Mayinga GP-MD pseudovirus infection at 1:300 serum dilutions as compared to sera from the Control Group (p < 0.01). In contrast, sera from GP-MN and GP-IM vaccinated mice were unable to neutralize EBOV-Mayinga GP-MD pseudoviruses as compared to sera from the Control Group (p > 0.1). Moreover, sera from GP-IM vaccinated mice were observed to enhance infectivity of EBOV-Mayinga GP-MD pseudoviruses at 1:900 serum dilutions compared to sera from the Control Group (p < 0.01). Taken together, these results indicate that neutralizing antibodies induced by immunization with unadjuvanted GP subunit vaccines seem to focus primarily on epitopes within the mucin-like domain of GP. However, in formulation with the Matrix-M adjuvant, immunization by GPadj-MN or GPadj-IM was able to induce high levels of neutralizing antibodies against epitopes in GP that are outside of the mucin-like domain.

### Immunization with GP subunit vaccines by MN patches confers more effective protection against lethal EBOV challenge than IM injection

**Figure 6:**
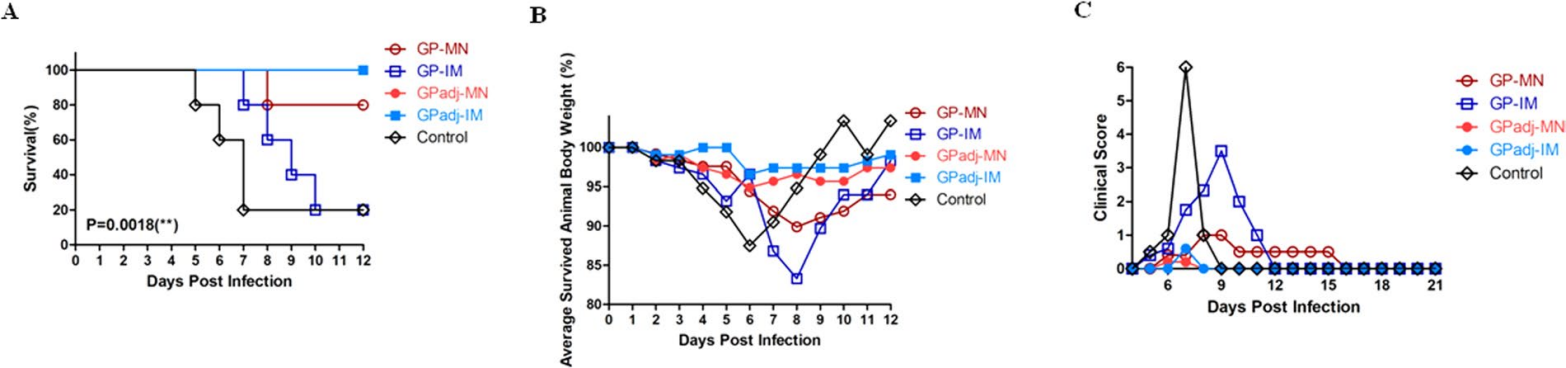
Protective efficacy against lethal EBOV challenge. Mice (groups of 5) were vaccinated twice at 4-week intervals by MN delivery (GPadj-MN) or IM injection (GPadj-IM) of GP nanoparticles in formulation with Matrix-M, or by MN delivery (GP-MN) or IM injection (GP-IM) of the same amount (5 µg) unadjuvanted GP nanoparticles. The control mice received IM injection of PBS. At 8 weeks after the second immunization, mice were challenged by intraperitoneal injection with 10^3^ plaque-forming units (pfu) of mouse-adapted Mayinga EBOV, and monitored daily for survival, body weight changes, and disease symptoms. (**A**) Daily survival rate of mice in each group post challenge. Statistical analysis of the Kaplan-Meier survival curves after challenge was conducted by Log-Rank analysis (p = 0.0018). (**B**) Average body weight of surviving mice in each group were determined daily post challenge and expressed as the percentage of the average body weight of the same group of mice on Day 0 of challenge. (**C**) Average daily clinical scores of surviving mice in each group post challenge. Clinical scores were recorded based on observation of for following symptoms: dyspnea (0–12), recumbency (0–12), responsiveness (0–12), appearance (0–3), eye appearance (0–3), nasal discharge (0–2), feed consumption (0–4), stool (0–1), and fluid intake (0–2), with “0” being normal and higher scores being more severe.

To compare the protective efficacy of different immunization approaches against EBOV infection, vaccinated mice were challenged at 8 weeks after the second immunization with 10^3^ plaque-forming units (pfu) of mouse-adapted EBOV-Mayinga, a heterologous EBOV strain that differs from the EBOV-Makona by 20 amino acids in GP sequence. Mice were monitored daily for 36 days after challenge to record weight changes, disease symptoms, and survival rates. As shown in Fig. 6A, only 1 of 5 mice in the GP-IM group survived the challenge, similar to the control group. Notably, deaths in the control group occurred from day 4 to 7 after challenge whereas deaths in the GP-IM group were delayed and occurred from day 7 to 10. In comparison, 4 of 5 mice in the GP-MN group survived the challenge; 1 animal died on day 8 post challenge. On the other hand, all mice in the GPadj-MN group and the GPadj-IM group that were vaccinated with adjuvanted GP subunit vaccines survived the challenge. The differences in survival rate between these groups were analyzed by Log-Rank analysis of the Kaplan-Meier survival curves and shown to be statistically significant (p = 0.0018). Monitoring of body weight changes of each group showed that a progressive loss in average body weight of surviving mice was observed for both the control and the GP-IM group, correlating with the timing of animal deaths in each group. In addition, loss of body weight was observed for mice in the GP-MN group, despite protection of 80% of mice from death in this group. In comparison, no significant group body weight change was detected for the GPadj-MN group and the GPadj-IM group. Comparison of disease symptoms post challenge showed that the clinical scores for the control group and the GP-IM group increased quickly after challenge, whereas the clinical score for the GP-MN group increased moderately on Day 8 post challenge and then dropped to a lower level after Day 10 post challenge. Further, only a transient and slight increase in clinical score was observed for mice in the GPadj-MN and GP-adj-IM groups. These results show that immunization by GP-MN conferred more effective protection against EBOV infection than immunization by GP-IM. Moreover, immunization with GP subunit vaccines in formulation with the Matrix-M adjuvant was able to confer complete protection against EBOV infection by either MN patch delivery or IM injection.

## Discussion

As a new vaccination technology, MN patches offer several advantages over conventional IM or SC injection methods by hypodermic needles with respect to vaccine stability, less pain during administration and thus increased acceptance, ease of use by self-administration, and elimination of biohazardous waste associated with hypodermic needle usage^17–20^. Over the past decade, MN patch-based delivery systems have been investigated for vaccination against a range of different diseases^26,27^, and have evolved rapidly from preclinical testing in animal models into clinical trials in recent years^22,28^. The focus of the present study is to investigate the utility of MN patches for vaccination with an EBOV GP subunit vaccine for the development of an effective and potentially thermally stable EBOV vaccine strategy.

Our results show that GP subunit vaccines can be successfully coated onto the solid-metal MN patches, and that the GP molecules retained their molecular integrity during the process of GP-MN preparation. By comparing immune responses induced by GP-MN vaccination and the conventional IM injection of the same dose of GP subunit vaccine in mice, we observed that immunization by GP-MN patches induced 4-fold higher levels of antibody responses against EBOV GP than IM injection. The antibody responses induced by GP-MN also exhibited higher levels of neutralizing activity against infection by Makona EBOV GP-mediated pseudovirus compared to GP-IM immunization. The skin system is rich in antigen presenting cells including Langerhans cells and dermal dendritic cells^29,30^. Vaccination by MN patches could deliver vaccine antigens directly to these antigen-presenting cells for stimulation of immune responses^31^. Some results from previous studies on MN patch delivery of inactivated influenza virus vaccines or influenza virus-like particles showed that MN patch vaccination induced similar levels of antibody responses against vaccine antigens as IM injection^27,32,33^. Here we found that MN patch delivery of EBOV GP nanoparticles induced increased antibody responses by 4-fold compared with IM injection of GP. These results indicate that immunization by MN patches may be more effective in boosting the immunogenicity of purified protein antigens. Moreover, the antibody responses against GP induced by GP-MN exhibit improved durability, with only about 30% reduction at 16 weeks after the second immunization. In contrast, the antibody responses against GP induced by IM injection declined by more than 3-fold during the same period. Results from previous studies have shown that inducing durable immune responses by adenovirus viral vector-based vaccines was important for providing long lasting protection against lethal EBOV challenge^34,35^. In recent clinical studies, it was reported that antibody levels peaked at 4 weeks after vaccination by adenovirus viral-vector based EBOV vaccines, leveled off at 8 weeks post vaccination with 2 to 4-fold decreases, and then remained stable until 48 weeks^36,37^. Therefore, it will be of interest to investigate whether sustained antibody responses could be induced by MN delivery of GP subunit vaccines in nonhuman primates or humans for providing long lasting protection against EBOV infection.

We also investigated whether immunogenicity of GP-MN vaccines can be further augmented by formulation with an adjuvant, Matrix-M, a saponin based adjuvant that has been shown to effectively augment immune responses induced by IM immunization with the GP subunit vaccine^23^. Our results showed that the GP in formulation with Matrix-M can also be coated onto solid metal MN patches, at comparable levels to GP subunit vaccine alone. Immunization studies in mice showed that vaccination by the adjuvanted GP-MN patches potently augmented induction of antibody responses against GP by about 8-fold compared to the unadjuvanted GP-MN patches, as similarly observed for IM injection. In further analysis of the IgG subclasses of antibody responses, we found that immunization by unadjuvanted GP nanoparticle vaccines by either MN patches or IM injection induced IgG1 but not IgG2a antibodies against GP. In contrast, immunization with adjuvanted GPadj-MN patches not only induced increased levels of IgG1 antibodies but also high levels of IgG2a antibodies against GP, similar to IM injection of the adjuvanted GP nanoparticles. Results from previous studies have shown that the saponin-based Matrix-M adjuvant may exert its adjuvant activity through activation of DCs and macrophages and recruitment of these cells to the draining lymph nodes^25^ and it is highly effective for augmenting induction of immune responses by various subunit vaccines via IM injection in both animal models and human clinical trials^38,39^. Our present results show that the Matrix-M retains its adjuvant activity when coated on MN patches, and is as effective to augment induction of both IgG1 and IgG2a antibodies in MN immunization as in IM immunization. In correlation with the induction of increased levels of antibodies against GP, sera from mice vaccinated by GPadj-MN or GPadj-IM exhibited higher levels of neutralizing activities against pseudoviruses containing GP from both the homologous Makona EBOV and the heterologous Mayinga EBOV GP compared to sera from mice vaccinated with unadjuvanted GP-MN or GP-IM. Further, sera from mice vaccinated by GPadj-MN or GPadj-IM also showed neutralizing activity against a pseudovirus containing a mucin-like domain-deleted GP from Mayinga EBOV (GP-MD), whereas sera from mice vaccinated with unadjuvanted GP-MN or GP-IM failed to neutralize this pseudovirus. These results suggest that the Matrix-M adjuvant also augmented induction of neutralizing antibodies against epitopes that are located outside of the mucin-like domain in GP.

The protective efficacies of various immunization regimens were evaluated against challenge by a mouse-adapted Mayinga EBOV, a heterologous EBOV strain that differs from the Makona EBOV by 20 amino acids in its GP sequence. Similar to the results from previous studies^23^, IM immunization with unadjuvanted GP failed to protect vaccinated mice against the lethal EBOV challenge, whereas IM immunization with adjuvanted GP provided complete protection. In comparison, immunization with the unadjuvanted GP-MN was able to protect 80% of vaccinated mice against the lethal EBOV challenge, and immunization with adjuvanted GPadj-MN was able to confer complete protection against challenge. Moreover, it was observed that mice vaccinated with adjuvanted GP subunit vaccines by either MN vaccination or IM injection only exhibited very slight and transient disease symptoms after challenge, indicating that these vaccination approaches were able to confer effective protection against both morbidity and mortality by caused by EBOV infection. Taken together, the present results demonstrate that MN patches represent an attractive approach for EBOV vaccine development, offering enhanced immunogenicity over IM injection. Further, delivery of GP subunit vaccines in dry forms by MN patches may improve its thermal stability as having been observed for influenza vaccines in previous studies^21,22^, and potentially overcome the cold-chain requirement for EBOV vaccine distribution. Further optimization of the MN vaccine delivery platform with respect to vaccine-adjuvant formulation and MN patch design and production will enable us to develop a safe, effective, and thermally stable vaccine against EBOV as well as other filoviruses.

## Methods

### Virus and biosafety

Mouse-adapted Mayinga EBOV stock was propagated in Vero E6 cells, and titered by a plaque assay before use for challenge studies^40^. All experiments involving infectious EBOV were performed at the biosafety level 4 (BSL-4) facility at the Texas Biomedical Research Institute.

### Cell lines, GP subunit vaccines, and adjuvant

293T cell and JC53 cells were maintained in Dulbecco’s Modified Eagle’s Medium (DMEM, Mediatech) supplemented with 10% fetal bovine serum (Hyclone, ThermoFisher) and 1% penicillin/streptomycin. Makona EBOV GP subunit vaccines were produced by Novavax Inc, in Sf9 insect cells using the recombinant baculovirus expression system under GMP conditions^23^. The purified Makona EBOV GP has been shown to assemble into spherical particles that are composed of GP trimers. The adjuvant, Matrix-M, is a proprietary adjuvant from Novavax, which is produced by formulating purified saponin with cholesterol and phospholipid^23^.

### MN patch preparation and characterization

MN patches were fabricated from stainless steel sheets and then treated by electropolishing using methods described previously^41^. Each MN patch contained a single row of five MNs. The individual MNs were measured to be 700 µm tall, with a cross-sectional area of 170 µm by 55 µm at the base and tapering to a sharp tip, and were prepared as described previously^27^. Makona EBOV GP nanoparticles with or without the Matrix-M1 adjuvant were concentrated by Vivaspin™ 500 filters (Sartorius™) of 30 K MWCO at a centrifugal force of 15000 × g for 30 min at 5 °C. Concentrated Makona EBOV GP nanoparticles with or without the Matrix-M1 adjuvant was then diluted (1:1) with the excipient solution (30% w/v trehalose and 2% w/v carboxymethyl cellulose sodium in phosphate buffer saline (PBS). Coating of MN patches was carried out using a similar dip-coating process as described previously^27^. To measure the amount of vaccine coated per MN patch, the vaccine coating on MN patches from each batch was dissolved into 200 µL of PBS. The protein concentration eluted in the solution was determined by a BCA protein assay (Pierce Biotechnology), and the quantity of vaccine antigen coated onto each MN patch was calculated. Vaccine antigens dissolved from MN patches were concentrated 10-fold using a protein concentrating column, and 1 µg of total protein were analyzed by SDS-PAGE followed by Western blot analysis in comparison with different amount of purified GP subunit vaccines. The amount of GP on MN patces was further determined by a quantitative ELISA. Briefly, vaccine antigens dissolved from MN patches were serially diluted and then used to coat a 96-well microtiter plate. In parallel, serial dilutions of purified GP with known concentrations were also coated onto the microtiter plate for generation of a standard curve. After coating, the wells were blocked by 5% BSA (bovine serum albumin) and the amount of GP coated in each wells of the plate was determined by ELISA using mouse-anti-GP antibodies (pooled sera from mice that had been vaccinated by EBOV GP DNA vaccines) as primary antibodies and HRP-conjugated goat-anti-mouse IgG antibodies as secondary antibodies. The amount of GP dissolved from MN patches was then calculated based on the standard curve generated using the purified GP.

### Immunization, blood sample collection, and challenge of mice

Eight-week-old female BALB/c mice (Charles River Laboratory) were housed at the Emory University animal facility in standard microisolator cages (5 mice per cage). All animal studies were carried out in accordance with relevant guidelines and regulations and approved by the Institutional Animal Care and Use Committees (IACUC) of Emory University, Georgia Institute of Technology, and the Texas Biomedical Research Institute.

Each mouse was vaccinated with purified GP protein (5 µg) with or without Matrix-M1 adjuvant (5 µg) via MN patches or IM injection. For immunization by MN patches, the hair on the stomach of the mouse skin was removed before vaccination by application of depilatory cream (Nair, Church & Dwight Co. Inc.). Under anesthesia by ketamine and xylazine, the mouse stomach skin was lightly stretched by hand, and MN patches were pressed into the skin and held in position for 2 min. For IM immunization, the same amount antigen was dissolved in 50 µL PBS and injected into the hind legs. Another group of mice receiving IM injection of 50 µL PBS was used as control.

For the first immunization study (results presented in Figs 1 and 2), 3 groups of mice were used with 5 mice per group, a group size that could provide 80% power to detect significant differences if the levels of immune responses differ by more than 2-fold between groups and vary less than 50% within each group. All mice were housed at the Emory University animal facility, vaccinated twice at 4-week intervals (Week 0 and Week 4). Immunization by MN patches were carried out by inserting 3 MN patches (5.4 ug total GP) in parallel to each other to the mouse skin at the same time. Blood samples were collected at 2 weeks after the second immunization (Week 6) as well as 16 weeks after the second immunization (Week 20). All blood samples were collected by facial bleeding without anesthesia in accordance with IACUC guidelines and protocols, heat inactivated, and stored at −80 °C until analysis.

For the second immunization study (results presented in Figs 3–6), 5 groups of mice were used with 5 mice in each group, a group size that could provide 80% power to detect significant differences if the survival rates between different groups differ by 80% or more. Mice were initially housed at the Emory University animal facility and vaccinated at Week 0 and Week 4, and blood samples were collected at Week 6. Immunization by GP-MN patches were carried out by inserting 6 MN patches (4.8 ug total GP) and immunization by GPadj-MN patches were carried out by inserting 8 MN patches (4.8 ug total GP) in parallel to each other to the mouse skin at the same time. After blood sample collection, vaccinated mice were shipped by certified courier to the Texas Biomedical Research Institute, and then challenged in its ABSL-4 facility at 8 weeks after the second immunization (Week 12). All mice were labeled with ear tags prior to shipment from Emory University, and the groups were randomly renamed to 1A, 1B, 2B, 2C, and 3C respectively (mice in each group were also individually marked with different colors on the tails) after entering into the ABSL-4 facility for blinded challenge. Mice were challenged by intraperitoneal injection with 10^3^ plaque-forming units (pfu) of mouse-adapted Mayinga EBOV. After challenge, mice were monitored for weight changes and signs of disease on a daily basis until day 36 post-challenge. Clinical scores were recorded based on observation of for following symptoms: dyspnea (0–12), recumbency (0–12), responsiveness (0–12), appearance (0–3), eye appearance (0–3), nasal discharge (0–2), feed consumption (0–4), stool (0–1), and fluid intake (0–2), with “0” being normal and higher scores being more severe. Mice with combined clinical scores over 12 were sacrificed by cervical dislocation under anesthesia based on IACUC endpoint. Mice that survived the challenge were sacrificed by cervical dislocation under anesthesia at the end of the study.

### ELISA

EBOV GP-specific antibodies in individual mouse serum samples were measured by ELISA, using established protocols^40,42–44^. Briefly, the assays were performed in a 96-well plate coated overnight at 4 °C with Makona EBOV GP proteins at concentration of 1 µg/ml and then blocked with 5% BSA. Serial dilutions of serum samples were incubated at room temperature for 2 hours on coated and blocked ELISA plates, and the bound immunoglobulins were detected with HRP-conjugated goat-anti-mouse IgG, IgG1 or IgG2a secondary antibodies (Southern Biotechnology Associates). The wells were developed with tetramethylbenzidine (Sigma). The color reaction was stopped with hydrochloric acid (0.2 N), and the absorbance at 450 nm was determined by an ELISA reader. A standard curve was constructed by coating each ELISA plate with serial 3-fold dilutions of purified mouse IgG, IgG1 or IgG2a antibodies with known concentrations, and the concentrations of GP-specific antibodies in serum samples were calculated using obtained standard curves and expressed as the mass (ng) of antigen-specific antibody per volume (ml) of serum sample.

### Pseudovirion neutralization assay

Neutralizing activity of sera from vaccinated mice was analyzed against pseudoviruses containing Makona EBOV GP, Mayinga EBOV GP, or a mucin-domain deleted Mayinga EBOV GP (GP-MD) as described in previous studies^42,44^. Briefly, 293T-cells were co-transfected with an Env-defective HIV backbone plasmid and a plasmid in which the genes for Makona EBOV GP, Mayinga EBOV GP, or Mayinga EBOV GP-MD were cloned in the pCAGGS vector. Supernatants were harvested 48 hours after transfection using Fugene HD (Roche), clarified, and filtered using a 0.45μm pore filter, and pseudoviruses were titered by infecting JC53 cells, which express β-galactosidase and luciferase under a *tat* activated promoter, causing infected cells to turn blue with X-Gal staining^45^. For determination of serum neutralizing activities, pseudoviruses (5 × 10^2^ pfu) were preincubated with serial 3-fold dilutions of heat-inactivated serum samples from each individual mouse and supplemented with heat-inactivated naive mouse sera (Innovative Research) so that 5% of the total volume was mouse serum. Pseudovirus-serum mixtures were then added to 50% confluent JC53 cells and incubated for 48 hours. Virus infection and neutralization was measured by a luciferase reporter assay, and neutralization was measured as the decrease in luciferase expression versus that for virus-naive mouse serum controls. Neutralizing activity is expressed as the percentage reduction of luciferase activity in sample wells, compared with luciferase activities in control wells with only naive mouse sera: [(luciferase activity in control well-luciferase activity in sample well)/(luciferase activity in control well)] × 100%.

### Statistics

The statistical significance for antibody levels and neutralizing activities between different groups was calculated by a two-tailed unpaired t-test and *p* ≤ 0.05 was considered significant. Statistical analysis of Kaplan-Meier survival curves was done by Log-Rank analysis and *p* ≤ 0.05 was considered significant.

### Data availability

The datasets generated during and/or analysed during the current study are included in this published article (and its Supplementary Information files).

## Electronic supplementary material

Supplementary Data

## References

[1] Mahanty S, Bray M (2004). Pathogenesis of filoviral haemorrhagic fevers. The Lancet. Infectious diseases.

[2] Shukarev G, Callendret B, Luhn K, Douoguih M, consortium E (2017). A two-dose heterologous prime-boost vaccine regimen eliciting sustained immune responses to Ebola Zaire could support a preventive strategy for future outbreaks. Human vaccines & immunotherapeutics.

[3] Lambe, T., Bowyer, G. & Ewer, K. J. A review of Phase I trials of Ebola virus vaccines: what can we learn from the race to develop novel vaccines? *Philosophical transactions of the Royal Society of London. Series B, Biological sciences* **372**, 10.1098/rstb.2016.0295 (2017). 10.1098/rstb.2016.0295 PMC5394635 28396468

[4] Warfield KL, Swenson DL, Demmin G, Bavari S (2005). Filovirus-like particles as vaccines and discovery tools. Expert review of vaccines.

[5] Hart MK (2003). Vaccine research efforts for filoviruses. International journal for parasitology.

[6] Weingartl HM (2012). Transmission of Ebola virus from pigs to non-human primates. Scientific reports.

[7] Sullivan NJ (2006). Immune protection of nonhuman primates against Ebola virus with single low-dose adenovirus vectors encoding modified GPs. PLoS medicine.

[8] Jones SM (2005). Live attenuated recombinant vaccine protects nonhuman primates against Ebola and Marburg viruses. Nature medicine.

[9] Bukreyev A (2007). Successful topical respiratory tract immunization of primates against Ebola virus. Journal of virology.

[10] Herbert AS (2013). Venezuelan equine encephalitis virus replicon particle vaccine protects nonhuman primates from intramuscular and aerosol challenge with ebolavirus. Journal of virology.

[11] Warfield KL (2007). Ebola virus-like particle-based vaccine protects nonhuman primates against lethal Ebola virus challenge. The Journal of infectious diseases.

[12] Swenson DL (2008). Vaccine to confer to nonhuman primates complete protection against multistrain Ebola and Marburg virus infections. Clinical and vaccine immunology: CVI.

[13] Wong G (2012). Immune parameters correlate with protection against ebola virus infection in rodents and nonhuman primates. Science translational medicine.

[14] Sullivan NJ, Martin JE, Graham BS, Nabel GJ (2009). Correlates of protective immunity for Ebola vaccines: implications for regulatory approval by the animal rule. Nature reviews. Microbiology.

[15] Martins KA, Jahrling PB, Bavari S, Kuhn JH (2016). Ebola virus disease candidate vaccines under evaluation in clinical trials. Expert review of vaccines.

[16] Henao-Restrepo AM (2015). Efficacy and effectiveness of an rVSV-vectored vaccine expressing Ebola surface glycoprotein: interim results from the Guinea ring vaccination cluster-randomised trial. Lancet.

[17] Prausnitz MR (2017). Engineering microneedle patches for vaccination and drug delivery to Skin. Annual review of chemical and biomolecular engineering.

[18] Shin CI, Jeong SD, Rejinold NS, Kim YC (2017). Microneedles for vaccine delivery: challenges and future perspectives. Therapeutic delivery.

[19] Marshall S, Sahm LJ, Moore AC (2016). The success of microneedle-mediated vaccine delivery into skin. Human vaccines & immunotherapeutics.

[20] Leone M, Monkare J, Bouwstra JA, Kersten G (2017). Dissolving microneedle patches for dermal vaccination. Pharmaceutical research.

[21] Chu LY (2016). Enhanced stability of inactivated influenza vaccine encapsulated in dissolving microneedle patches. Pharmaceutical research.

[22] Rouphael NG (2017). The safety, immunogenicity, and acceptability of inactivated influenza vaccine delivered by microneedle patch (TIV-MNP 2015): a randomised, partly blinded, placebo-controlled, phase 1 trial. Lancet.

[23] Bengtsson KL (2016). Matrix-M adjuvant enhances antibody, cellular and protective immune responses of a Zaire Ebola/Makona virus glycoprotein (GP) nanoparticle vaccine in mice. Vaccine.

[24] Yang, H. W. *et al*. Ebola vaccination using a DNA vaccine coated on PLGA-PLL/gammaPGA nanoparticles ddministered using a microneedle patch. *Advanced healthcare materials* **6**, 10.1002/adhm.201600750 (2017). 10.1002/adhm.201600750 28075069

[25] Magnusson SE (2013). Immune enhancing properties of the novel Matrix-M adjuvant leads to potentiated immune responses to an influenza vaccine in mice. Vaccine.

[26] Matsuo K (2014). Vaccine efficacy of transcutaneous immunization with amyloid beta using a dissolving microneedle array in a mouse model of Alzheimer’s disease. Journal of neuroimmunology.

[27] Zhu Q (2009). Immunization by vaccine-coated microneedle arrays protects against lethal influenza virus challenge. Proceedings of the National Academy of Sciences of the United States of America.

[28] Hirobe S (2015). Clinical study and stability assessment of a novel transcutaneous influenza vaccination using a dissolving microneedle patch. Biomaterials.

[29] Miller LS, Modlin RL (2007). Toll-like receptors in the skin. Seminars in immunopathology.

[30] Kupper TS, Fuhlbrigge RC (2004). Immune surveillance in the skin: mechanisms and clinical consequences. Nature reviews. Immunology.

[31] del Pilar Martin M (2012). Local response to microneedle-based influenza immunization in the skin. mBio.

[32] Song JM (2010). Microneedle delivery of H5N1 influenza virus-like particles to the skin induces long-lasting B- and T-cell responses in mice. Clinical and vaccine immunology: CVI.

[33] Sullivan SP (2010). Dissolving polymer microneedle patches for influenza vaccination. Nature medicine.

[34] Stanley DA (2014). Chimpanzee adenovirus vaccine generates acute and durable protective immunity against ebolavirus challenge. Nature medicine.

[35] Sullivan NJ (2011). CD8+ cellular immunity mediates rAd5 vaccine protection against Ebola virus infection of nonhuman primates. Nature medicine.

[36] Ledgerwood JE (2017). Chimpanzee adenovirus vector Ebola vaccine. The New England journal of medicine.

[37] Zhu FC (2017). Safety and immunogenicity of a recombinant adenovirus type-5 vector-based Ebola vaccine in healthy adults in Sierra Leone: a single-centre, randomised, double-blind, placebo-controlled, phase 2 trial. Lancet.

[38] Morelli AB (2012). ISCOMATRIX: a novel adjuvant for use in prophylactic and therapeutic vaccines against infectious diseases. Journal of medical microbiology.

[39] Drane D, Gittleson C, Boyle J, Maraskovsky E (2007). ISCOMATRIX adjuvant for prophylactic and therapeutic vaccines. Expert review of vaccines.

[40] Sun Y (2009). Protection against lethal challenge by Ebola virus-like particles produced in insect cells. Virology.

[41] Gill HS, Prausnitz MR (2007). Coated microneedles for transdermal delivery. Journal of controlled release: official journal of the Controlled Release Society.

[42] Ye L (2006). Ebola virus-like particles produced in insect cells exhibit dendritic cell stimulating activity and induce neutralizing antibodies. Virology.

[43] Li W (2015). Characterization of immune responses induced by Ebola virus glycoprotein (GP) and truncated GP isoform DNA vaccines and protection against lethal Ebola virus challenge in mice. The Journal of infectious diseases.

[44] Mohan GS, Li W, Ye L, Compans RW, Yang C (2012). Antigenic subversion: a novel mechanism of host immune evasion by Ebola virus. PLoS pathogens.

[45] Wei X (2003). Antibody neutralization and escape by HIV-1. Nature.

