# Author Correction: Intradermal immunization by Ebola virus GP subunit vaccines using microneedle patches protects mice against lethal EBOV challenge

**DOI:** 10.1038/s41598-023-40413-0

**Published:** 2023-08-22

**Authors:** Ying Liu, Ling Ye, Fang Lin, Yasmine Gomaa, David Flyer, Ricardo Carrion, Jean L. Patterson, Mark R. Prausnitz, Gale Smith, Gregory Glenn, Hua Wu, Richard W. Compans, Chinglai Yang

**Affiliations:** 1 Key Laboratory of Special Animal Epidemic Disease, Ministry of Agriculture of China, Institute of Special Economic Animals and Plants, Chinese Academy of Agricultural Sciences CAAS, Changchun, Jilin 130112 P. R. China; 2 Emory University School of Medicine, 1518 Clifton Road, Atlanta, GA 30322 USA; 3 Central Laboratory, Tangdu Hospital at the Fourth Military Medical University, Xi’An, 710038 China; 4 Georgia Institute of Technology, 311 Ferst Drive, Atlanta, GA 30332 USA; 5 Department of Pharmaceutics, Faculty of Pharmacy, Alexandria University, El-Khartoum Square, Alexandria, 21521 Egypt; 6 Novavax Inc., 20 Firstfield Road, Gaithersburg, MD 20878 USA; 7 Texas Biomedical Research Institute, 7620 NW Loop 410, San Antonio, TX 78227 USA

Correction to: *Scientific Reports* 10.1038/s41598-018-29135-w, published online 25 July 2018

This Article and the accompanying Supplementary Data file contain an error in the affiliations, where an affiliation was inadvertently omitted for author Yasmine Gomaa.

Their correct affiliations are listed below.

Georgia Institute of Technology, 311 Ferst Drive, Atlanta, GA, 30332, USA.

Department of Pharmaceutics, Faculty of Pharmacy, Alexandria University, El-Khartoum Square, Alexandria, 21521, Egypt.

The corrected Supplementary Information file is linked to this correction notice.

### Supplementary Information

Supplementary Information.

