# Supplementary material for: Intradermal immunization by Ebola virus GP subunit vaccines using microneedle patches protects mice against lethal EBOV challenge
Source: Sci Rep. 2018 Jul 25;8:11193. doi: 10.1038/s41598-018-29135-w (PMC6060117; doi:10.1038/s41598-018-29135-w)
Supplement: Supplementary file 1 — Supplementary Data [file 41598_2018_29135_MOESM1_ESM.docx]

**Intradermal immunization by Ebola virus GP subunit vaccines using microneedle patches protects mice against lethal EBOV challenge**

Ying Liu^1,2^, Ling Ye^2^, Fang Lin^2,3^, Yasmine Gomaa^4^, David Flyer^5^, Ricardo Carrion Jr.^6^, Jean L Patterson^6^, Mark R. Prausnitz^4^, Gale Smith^5^, Gregory Glenn^5^, Hua Wu^1^, Richard W. Compans^2^, Chinglai Yang^2,*^,

^1^Key Laboratory of Special Animal Epidemic Disease, Ministry of Agriculture, P. R. China, Institute of Special Economic Animals and Plants, Chinese Academy of Agricultural Sciences CAAS, Changchun 130112, Jilin, China; ^2^Emory University School of Medicine, 1518 Clifton Road, Atlanta, GA 30322, USA; ^3^Central Laboratory, Tangdu Hospital at the Fourth Military Medical University, Xi'An 710038, China; ^4^ Georgia Institute of Technology, 311 Ferst Drive, Atlanta, GA 30332, USA; ^5^Novavax Inc., 20 Firstfield Road, Gaithersburg, MD 20878, USA; ^6^Texas Biomedical Research Institute, 7620 NW Loop 410, San Antonio, TX 78227, USA.

*To whom correspondence and proofs should be sent.

Dr. Chinglai Yang, PhD

Department of Microbiology and Immunology

Emory University School of Medicine

1518 Clifton Road, Room 5053 CNR

Atlanta, GA 30322, USA

Tel.: (1) 404-712-9607; FAX: (1) 404-727-3295; email: chyang@emory.edu

**Supplementary Information:**

**Suppl. Figure 1.**

|  |
| --- |
| **Supplementary Figure 1A. Characterization of GP subunit vaccines coated on MN by** **ELISA.** GP subunit vaccines were coated onto MN patches as described in Materials and Methods. Five patches were randomly selected from the production batch and GP proteins were dissolved from MN patches by incubating the MN patches in 200 µl PBS, to determine the amount of GP from each MN patch by a quantitative ELISA using sera from mice vaccinated by EBOV GP DNA vaccines. GP proteins dissolved from each GP-MN were coated onto the wells of a 96-well plate in triplicates. Serial dilutions of purified GP proteins were also coated onto the wells of the 96-well plate at known concentrations to generate a standard curve as shown. The amount of GP dissolved from GP-MN was determined by ELISA based on a standard curve generated with purified GP with known concentrations. |

| 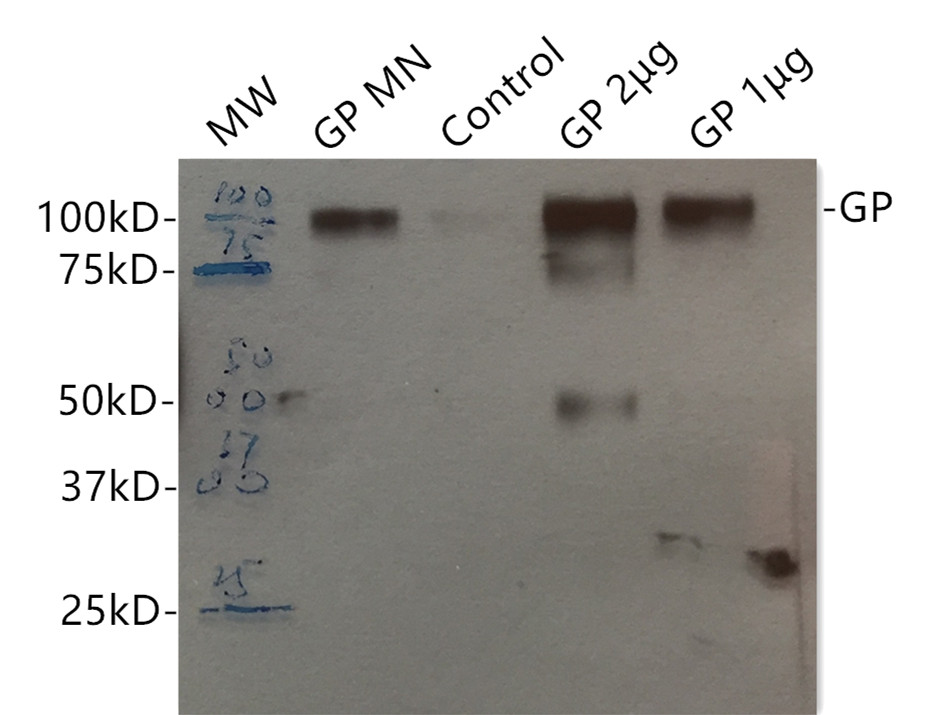 |
| --- |
| **Supplementary Figure 1B. Characterization of GP subunit vaccines coated on MN by** **Western blot.** GP proteins dissolved from GP-MN were concentrated and the protein concentration was determined by a BCA assay. The dissolved GP proteins (1 µg) were analyzed by SDS-PAGE and Western blot in comparison with 1 µg and 2 µg purified GP proteins. MW, molecular weight marker. GP MN, GP (1 µg) dissolved from MN patches. Control, PBS only. GP 2µg, 2 µg of purified GP. GP 1 µg, 1 µg of purified. |

**Suppl. Figure 2.**

|  |
| --- |
| **Supplementary Figure 2A. Characterization of GP subunit vaccines coated on MN by** **ELISA.** GP subunit vaccines were formulated with Matrix-M adjuvant at a 1:1 ratio (amount of GP vs. amount of saponin) and then coated onto MN patches as described in Materials and Methods. GP-MN were prepared by mixing GP with the same volume of PBS prior to coating onto MN patches. Five patches were randomly selected from the production batch and GP proteins were dissolved from GPadj-MN and GP-MN patches by soaking the patches in 200 µl PBS, to determine the amount of GP from each MN patch by a quantitative ELISA using sera from mice vaccinated by EBOV GP DNA vaccines. GP proteins dissolved from each GP-MN were coated onto the wells of a 96-well plate in triplicates. Serial dilutions of purified GP proteins were also coated onto the wells of the 96-well plate at known concentrations to generate a standard curve as shown. The amount of GP dissolved from GP-MN or GPadj-MN was determined by ELISA based on a standard curve generated with purified GP with known concentrations. |

| **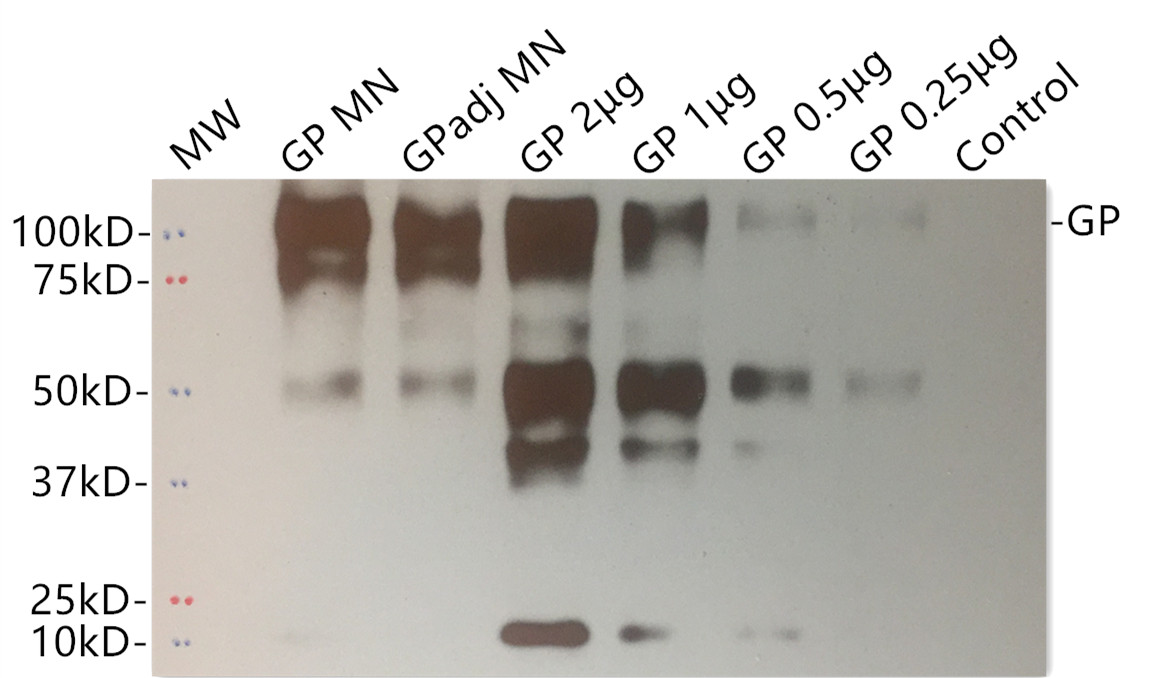** |
| --- |
| **Supplementary Figure 2B. Characterization of GP subunit vaccines in formulation with Matrix-M coated on MN by** **Western blot.** GP proteins dissolved from GPadj-MN or GP-MN were concentrated and the protein concentration was determined by a BCA assay. The dissolved GP proteins (1 µg) were analyzed by SDS-PAGE and Western blot in comparison with 0.25 µg, 0.5 µg, 1 µg, and 2 µg purified GP nanoparticles. MW, molecular weight marker. GP MN, GP (1 µg) dissolved from GP-MN patches. GPadj MN, GP (1 µg) dissolved from GPadj-MN patches . GP 2µg, 2 µg of purified GP. GP 1 µg, 1 µg of purified GP. GP 0.5µg, 0.5 µg of purified GP. GP 0.25 µg, 0.25 µg of purified GP. Control, PBS only. |
